# Activation of AMPK under Hypoxia: Many Roads Leading to Rome

**DOI:** 10.3390/ijms21072428

**Published:** 2020-03-31

**Authors:** Franziska Dengler

**Affiliations:** Institute of Veterinary Physiology, University of Leipzig, D-04103 Leipzig, Germany; dengler@vetmed.uni-leipzig.de; Tel.: +49-341-97-38-073

**Keywords:** AMPK, [Ca^2+^_i_], CaMKK, FIH1, HIF, HIF-P4H, hypoxia, LKB1, reactive oxygen species (ROS)

## Abstract

AMP-activated protein kinase (AMPK) is known as a pivotal cellular energy sensor, mediating the adaptation to low energy levels by deactivating anabolic processes and activating catabolic processes in order to restore the cellular ATP supply when the cellular AMP/ATP ratio is increased. Besides this well-known role, it has also been shown to exert protective effects under hypoxia. While an insufficient supply with oxygen might easily deplete cellular energy levels, i.e., ATP concentration, manifold other mechanisms have been suggested and are heavily disputed regarding the activation of AMPK under hypoxia independently from cellular AMP concentrations. However, an activation of AMPK preceding energy depletion could induce a timely adaptation reaction preventing more serious damage. A connection between AMPK and the master regulator of hypoxic adaptation via gene transcription, hypoxia-inducible factor (HIF), has also been taken into account, orchestrating their concerted protective action. This review will summarize the current knowledge on mechanisms of AMPK activation under hypoxia and its interrelationship with HIF.

## 1. Introduction

The AMP-activated protein kinase (AMPK) is a well-known energy sensor, not only in mammalian cells, but has been shown to be an ancient mechanism that has been conserved throughout the evolution of complex life [1,2,3]. In the last decades its upstream and downstream regulatory mechanisms have been characterized [2], and therapeutic implications of its activation and inhibition, respectively, are beyond speculation [4,5]. However, besides its classical role as an energy sensor, not all aspects of its biological function have yet been completely unraveled.

Hypoxia, i.e., an inadequate oxygen supply compared to the cellular demand, occurs not only in pathological situations like ischemic stroke or myocardial ischemia, but is a common feature under inflammatory conditions and also perceived in healthy organs depending on their current perfusion rate [6]. Thus, there seem to be adaptation mechanisms to hypoxia in order to secure cellular survival under these conditions.

An activation of AMPK under hypoxia has been reported repeatedly while the mechanism of this activation and its biological relevance are under discussion. This review will focus on summarizing the current knowledge regarding the role and activation of AMPK under hypoxia.

## 2. General Characteristics of AMPK

The serine/threonine kinase AMPK is composed of three subunits (α, β and γ) [7]. Each of these subunits comprises several isoforms (so far α1, α2, β1, β2, γ1, γ2, γ3); thus, a total of at least 12 combinations and thus isoenzymes are possible. These isoenzymes seem to have different tissue distributions and also specific functions, which are just in the early stages of classification [8]. However, all heterotrimers are basically activated by phosphorylation of a threonine residue within the catalytic α subunit [3,7]. This residue is conformally called “Thr172”, according to its position in the human sequence, although it might vary between species. Besides this phosphorylation, two other mechanisms enhance AMPK activation. Binding of AMP (and to a lesser extent also ADP) to the regulatory γ subunit leads to i) an allosteric activation supporting phosphorylation at the α subunit and ii) prevents the dephosphorylation of Thr172 [2], thus coupling its activity tightly to cellular energy levels. The phosphorylation at Thr172 is mediated by at least two upstream kinases. The first to be identified was liver kinase B1 (LKB1) [1,9], while identification of Ca^2+^/calmodulin-dependent protein kinase (CaMKK) 2, also known as β, as another upstream kinase followed soon after [10,11,12]. There are hints that they might even exert different isoform-specific effects on AMPK [13]. There might still be other kinases that activate AMPK but are not characterized yet [14]. Thus, transforming growth factor-β-activated kinase 1 (TAK1) might also phosphorylate AMPK at Thr172 under specific conditions like inflammation [15,16].

The activation of AMPK has mainly been reported to be initiated by an increased AMP/ATP ratio, i.e., when cellular energy levels drop due to cellular stresses like glucose deprivation, inhibition of mitochondrial oxidative phosphorylation or toxic agents [3,17]. Then, it acts on both gene expression and protein levels to adapt the cellular metabolism by deactivating energy consuming, anabolic processes and activating processes that deliver “cheap” energy such as glycolysis [2,18]. AMPK has also been shown to be a major promotor of mitochondrial biogenesis, thus enhancing cellular capability to provide energy substrates [19]. So far, more than 60 targets of AMPK have been identified [3]. Numerous extensive reviews on the targets and characteristics of AMPK can be found [20,21,22], while this review will focus on the role of AMPK activation under hypoxia.

## 3. Definition of Hypoxia

Hypoxia is defined as a condition in which the cellular oxygen supply is reduced so that it does not meet the cellular oxygen requirements [23]. Due to variations in tissue perfusion, oxygen levels may change throughout the day and with the functional status of the tissue, whereas more oxygen-sensitive tissues like the brain or the heart need a constantly high oxygen supply and quickly suffer from oxygen deprivation (e.g., an ischemic stroke) [24]. This disease pattern infers another problem: due to its origin in a lack of perfusion, hypoxia is often accompanied by a lack of nutrients as well, termed ischemia. Therefore, it is hard to judge mechanisms initiated by hypoxia independently from the cellular energy status.

However, hypoxia is not always associated with severe pathologies like ischemic stroke or myocardial infarction, but it also occurs under inflammatory conditions [6] and within the physiological range of the cells’ adaptation capacities, being referred to as “physiological hypoxia” [23]. The intestinal epithelium gives a great example of this circumstance because of its location as the borderline between the anaerobic gut lumen and the organism, which renders its oxygen supply to the blood vessels only at the basal cell pole. Additionally, this perfusion underlies large variations reflecting the digestive activity and thus usually leaves the enterocytes with a mere 2−4% oxygen level [25]. Still, these cells function well with this varying perfusion, implicating that they are equipped with effective adaptation mechanisms to survive under hypoxia. AMPK might play a central role in mediating this adaptation.

## 4. The Role of AMPK under Hypoxia

It is intriguing that saving energy is an important rescue factor in cells undergoing hypoxia. Therefore, a role of AMPK in hypoxic adaptation seems logical. Indeed, there has been increasing evidence of activation of AMPK acting protectively in hypoxia-associated pathologies, e.g., ischemic injury in heart, brain or guts [26,27,28,29]. By decreasing the cellular ATP consumption, the oxygen demand is also lowered; thus, cellular survival is enhanced [30,31].

An activation of AMPK under hypoxia has been demonstrated in various tissues and cell types, ranging from brain [32,33,34], liver [35] and skeletal muscle [36,37,38] to cardiomyocytes [39,40], alveolar epithelial cells [41,42], intestinal epithelial cells [43,44] and fibroblasts [45]. Interestingly, an inhibition of AMPK activation under hypoxia was reported after long-term hypoxia (in this case 8% oxygen for 12 d) [46]. However, in the latter study mice were not only kept under hypoxia for a long period but also fasted 24 h after that, which might explain discrepancies to other studies. Older mice have also been reported to have a blunted response of AMPK to hypoxia, which might be explained by already constitutively increased levels of AMPK activation in comparison to younger animals [35]. In contrast, a suppression of AMPK activity was observed under hyperoxia [47], forming the counterpart to hypoxia-mediated activation.

An important mechanism to secure cellular survival under hypoxia is autophagy [48], which has been demonstrated to be initiated by AMPK in various cell types [49,50,51,52]. This further strengthens the assumption that AMPK plays a major role in the adaptation to hypoxic stress. Although a few studies also observed AMPK-independent initiation of autophagy under hypoxia [53,54], nowadays AMPK is well accepted as a crucial factor for cellular survival during and in recovery after hypoxic insults. The mode of its action and activation, though, is not yet as clear. With respect to the coupling of hypoxia to metabolism, the increased cellular AMP levels induced by hypoxia may be considered the only pathway inducing the activation of AMPK under hypoxia. However, an activation of the adaptation mechanisms even before the cells enter the actual emergency would be desirable. Thus, a lot of research has been conducted aiming to identify other mechanisms activating AMPK under hypoxia. In the following, the current knowledge on the activation of AMPK with special consideration of hypoxia will be summarized.

## 5. Activation of AMPK under Hypoxia

While activation of AMPK under hypoxia has often been shown, it is still under discussion whether this activation is mediated by the hypoxic stimulus specifically [55,56] or if it is a side effect of the metabolic (and thus energetic) consequences inferred by hypoxia [47,57]. Also, if the former was true, how is the activation of AMPK mediated under hypoxia?

**Figure 1:**
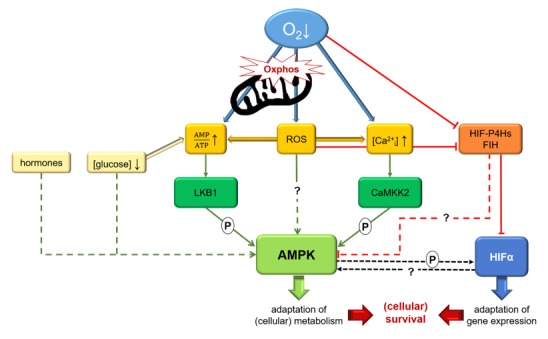
Graphical summary illustrating possible mechanisms regulating AMP-activated protein kinase (AMPK) activity under hypoxia. A decreased oxygen supply impairs oxidative phosphorylation (oxphos) in mitochondria and thus leads to increased AMP/ATP ratios, which will activate the canonical pathway of AMPK activation by LKB1. Besides LKB1, AMPK can also be phosphorylated by CaMKK2 subsequently to an increase of [Ca^2+^_i_] under hypoxia. Impaired mitochondrial oxphos also causes formation of reactive oxygen species (ROS), which might not only induce these pathways but also activate AMPK directly. ROS are also supposed to inhibit hypoxia-inducible factor (HIF)-hydroxylase enzymes, which work oxygen-dependently and have been suggested to regulate AMPK in concert with HIF. Crosstalk between AMPK and HIF might help to orchestrate the adaptation to low oxygen levels as well. More details are given in the text.

It is beyond doubt that the upstream kinases LKB1 and CaMKK2 play a central role in phosphorylating and thus activating AMPK [58]. Still, their activation has to be initiated, and there may additionally be other pathways independent of the upstream kinases [59,60]. Besides the canonical pathway via an increased AMP/ATP ratio with subsequent activation of LKB1, there is proof of a Ca^2+^-dependent activation of AMPK by CAMKK2 under hypoxia. Many studies claim that an increased production of reactive oxygen species (ROS) under hypoxia leads to an activation of AMPK, while hypoxia-inducible factor (HIF)-hydroxylase enzymes have been supposed to play a role in the regulation of AMPK, but there is little proof. Finally, several hormones and systemic glucose levels seem to play a role as well. In the following, the mechanisms proposed and investigated so far will be presented. They are summarized in Figure 1.

### 5.1. AMP

The canonical way of activating AMPK via an increased AMP/ATP ratio has been demonstrated under hypoxic conditions [42,61]. This makes sense, as a depletion of oxygen would always lead to a restrained metabolism, especially mitochondrial oxidative phosphorylation, resulting in low cellular energy levels. An AMP-dependent activation of AMPK was reported in lung epithelial cells under hypoxia [42] and in kidney epithelial cells under ischemia [27] as well as in murine heart muscle [62]. Specialized oxygen-sensing cells in the carotid body seem to react to an increased AMP/ATP ratio as well [63], indicating that AMPK is not only guarding cellular but also systemic oxygen levels.

However, there have also been many reports of an activation of AMPK without any observable changes in AMP concentrations [41,59,64]. It would be advantageous if the cellular adaptation machinery was activated even before the actual damage happens. In contrast to that, Wilson et al. argue that total AMP concentration, which is usually measured in cultured cells, does not equal free (and more relevant in terms of energy supply) AMP concentration, which might therefore be underestimated in many studies [47]. There is evidence that the different isoforms of the AMPK subunits display different sensitivities to AMP and might thus react to different stimuli [8] and imply different sensitivities according to their tissue distribution.

### 5.2. LKB1 versus CaMKK2

It has been shown that activation of AMPK via increased AMP/ATP ratios primarily implies activation via LKB1. This conclusion is suggested by studies in mice with a hypomorphic expression of LKB1, which abrogated AMPK activation under hypoxia in smooth muscle cells, while a knockout of CaMKK2 had no effect on the activation of AMPK under hypoxia in mice [57]. In lung epithelial cells, only inhibition of LKB1, but no other upstream kinase, abrogated the activation of AMPK under hypoxia [42].

In contrast, CaMKK2 seemed to be responsible for AMPK activation under hypoxia in alveolar epithelial cells [39]. This might be explained by different abundances of the upstream kinases in these cell types [42], but also by differing specificities for the AMPK subunit isoforms. Thus, LKB1 seems to activate only the α2 but not α1 subunit in muscle cells [13,62]. However, an activation of both subunits was observed under hypoxia in the heart muscle of mice, and the α1 subunit was phosphorylated in LKB1-deficient cells as well [62], indicating that another upstream kinase besides LKB1 activates AMPK under hypoxia; CaMKK2 being the natural candidate.

### 5.3. CaMKK2 and Intracellular [Ca^2+^]

Several groups report a connection of AMPK activation under hypoxia and [Ca^2+^_i_] [32,65,66]. The most obvious explanation would be a Ca^2+^-dependent activation of the upstream kinase CaMKK2. This was shown in both HeLa and HEK293T cells where AMPK was activated under hypoxia concurrently with an increase of [Ca^2+^_i_] and inhibited by incubation with the CaMKK2 inhibitor STO-609 [67]. It was demonstrated in alveolar epithelial cells (in contrast to lung epithelial cells, see above) that, subsequently to hypoxia, an increase in [Ca^2+^_i_] led to a CaMKK2-dependent activation of AMPK [42]. Thus, the regulation of AMPK activity under hypoxia might differ tissue-dependently.

### 5.4. Reactive Oxygen Species

H_2_O_2_, O_2_^-^ and HO are summarized under the term ROS [68,69]. While the single members of this group vary greatly in their roles and effects, all of them act as intracellular messengers both in microorganisms and in mammals [68,70]. There are ambivalent reports on the formation of ROS under hypoxia: while the production of ROS might be reduced in order to avoid cellular damage, it could also be increased as part of the oxygen-sensitive signaling system mediating the adaptation reaction [69]. These contradicting findings may be due to diverse model systems used and are discussed in recent reviews [69,70].

It has been demonstrated, though, that ROS formation is accompanied by an activation of AMPK [60,71,72] and that the ROS are generated by mitochondrial complex III under hypoxia [70,71]. This ROS-dependent activation was abrogated by the additional incubation with ROS scavengers [55]. In contrast, Tan et al. could not confirm any effect of the scavengers on activation of AMPK under hypoxia, e.g., in airway epithelial cells [42].

Additionally, the reports vary in the means by which this ROS-dependent activation of AMPK is supposed to work. Due to the harmful accumulation of ROS, the AMP/ATP ratio could be increased, thus leading to an activation of AMPK [73,74], whereas others report the AMP concentration to be unchanged after hypoxia-induced ROS formation and AMPK activation [41,55,60,71]. Still, there are different theories on AMP-independent mechanisms downstream of ROS: Zmijewski et al. [60] propose the oxidation of a cysteine residue in the AMPKα subunit by H_2_O_2_, leading to its activation, partly by autophosphorylation; Emerling et al. [71] demonstrate that the ROS-dependent activation of AMPK is necessarily mediated by LKB1 and might be seconded to a small part by CaMKK2; and last but not least, Mungai et al. [55] and Gusarova et al. [41,75] find a coupling to [Ca^2+^_i_], which would then activate AMPK via CaMKK2.

Besides ROS, also CO_2_ [76] and CO [66] seem to induce an activation of AMPK in alveolar epithelial cells and astrocytes.

However, AMPK seems to be involved in cellular redox signaling in both directions, which is mirrored by its influence on mitochondrial biogenesis, the site of both production and elimination of ROS [19,77]. Thus, an activation of AMPK has been shown to stabilize cellular ROS production below a toxic threshold [77]. Additionally, there seems to be a functional triangle between AMPK, the mammalian target of rapamycin complex (mTORC) 1 and thioredoxin-interacting protein (TXNIP) in a way that AMPK controls autophagy and excessive production of ROS mediated by mTORC1 and TXNIP [78]. The involvement of AMPK in cellular redox homeostasis has been reviewed extensively elsewhere [77].

### 5.5. HIF-hydroxylase Enzymes

Being subject of the latest Nobel Prize in Physiology or Medicine, the hypoxia-inducible factor (HIF) is well known as the master regulator of gene transcription under hypoxia [79,80]. Its regulation by hydroxylase enzymes has been thoroughly characterized by the Nobel laureates (extensively reviewed in [81]) and is mainly mediated by hydroxylation at proline residues by the HIF-prolyl-4-hydroxylases (HIF-P4Hs) and additionally by asparaginyl hydroxylation by another hydroxylase termed factor inhibiting HIF1α (FIH1) [82]. So far, the HIF-P4Hs occur in four isoforms (HIF-P4H 1, 2, 3 and transmembrane). This oxygen-dependent hydroxylation leads to the degradation of the HIFα subunit in the cytoplasm, thus preventing its translocation into the nucleus where it would dimerize with the β subunit and lead to the transcription of >300 genes mediating cellular adaptation to hypoxia. Under hypoxia, the hydroxylation is hampered due to the lack of the cofactor oxygen, and the translocation and thus transcription is initiated [83].

Therefore, it would be convenient if AMPK was regulated by the same hydroxylation mechanisms under hypoxia, orchestrating a common adaptation reaction. Indeed, we could previously show an activation of AMPK in CaCo-2 cells both under hypoxia and upon treatment with the pan-hydroxylase inhibitor dimethyloxalylglycine (DMOG) [44]. In line, Yan et al. [84] were able to induce an activation of AMPK in cardiomyocytes by application of DMOG or siRNA for HIF-P4H 2. This activation was dependent on [Ca^2+^_i_] and CaMKK (see above), but not on LKB1 [84]. Thus, the authors propose a coupling of HIF-P4Hs and [Ca^2+^_i_], leading to an activation of AMPK under hypoxia [84].

In contrast, a group of renowned researchers recently reported that they could not confirm any other speculated hydroxylation targets of the HIF-P4Hs than HIF, casting the role of HIF-P4Hs in the regulation of AMPK into doubt [85]. FIH1, though, has a more accessible catalytic site and, unlike the HIF-P4Hs, does not undergo major conformational changes upon substrate binding and may thus be less substrate-specific [85]. Activation of AMPK by FIH1 has already been described in brown adipose tissue [56], although proof of an actual hydroxylation of AMPK is still lacking. Scholz et al. [86] suggest an indirect action of FIH1 on AMPK activity via the deubiquitinase OTUB1 in human embryonic kidney cells.

### 5.6. Hormones and Other Mediators

The protective effects of AMPK have been considered in the context of several hormones that might be associated with (systemic) energy depletion or inflammatory stimuli. The “hunger hormone” ghrelin has been considered to be protective in ischemia/reperfusion injury and has been shown to activate AMPK [87,88], probably via CaMKK2 [89]. Also, leptin signaling, which basically acts antagonistic to ghrelin, has been reported to activate AMPK in chronic intermittent hypoxia independently of AMP levels in peripheral tissues like skeletal muscle [13,64,90], whereas it exerts an inhibitory effect on hypothalamic AMPK [91,92]. This could be interpreted as another sign of the tissue-specific roles of AMPK, controlling both local and systemic energy levels. The systemic metabolic signaling might not be connected directly with hypoxia, though, but rather considered as an effect of a general metabolic switch that is most probably started by energetic imbalances.

Besides this more “obvious” connection, there have also been reports about the coagulation enzyme thrombin and its antagonist antithrombin to act protectively under hypoxia via activation of AMPK [59,93]. With respect to thrombin, this seems to be mediated via G-protein coupled receptors leading to an increase of [Ca^2+^_i_] and thus an activation of CaMKK2 [59], whereas antithrombin seems to apply an inflammatory signaling pathway [93].

### 5.7. Glucose and Coupling to mTORC1

Another non-canonical way of activating AMPK is glucose-sensing by a mechanism that was termed the “lysosomal pathway” [22,94]. Although not directly connected to hypoxia, it is still worth mentioning because an impaired perfusion infers not only a lack of oxygen but also glucose (i.e., ischemia). As a lack of glucose might also quickly result in an increased AMP/ATP ratio, it is all the more interesting that there also seem to be cellular mechanisms anticipating a lack of glucose even before it might become a problem in terms of energy levels.

Thus, aldolase has been proposed as a link between the availability of substrates and the activation of AMPK by an aldolase–vATPase–Ragulator complex at the lysosomal membrane, which will additionally bind AXIN-LKB1 and then activate AMPK at low cellular glucose concentrations [40,94,95]. At high glucose concentrations, this aldolase–vATPase–Ragulator complex is associated with the activation of mTORC1. Thus, the lysosomal pathway seems to be a converging point of AMPK and mTORC1, that have long ago been shown to act antagonistic, for example, in terms of autophagy, oxidative phosphorylation (and thus generation of ROS) and protein anabolism [96]. While an inhibition of the mTOR pathway by AMPK has been described before [97], this spatial connection has only been discovered recently [40,94] and might also imply a fine-tuned coordination of these pathways. With respect to the role both mechanisms play in the cellular adaptation to hypoxia [98], this coordination could also be beneficial under hypoxic conditions.

## 6. Crosstalk between AMPK and HIF

Due to their common protective role under hypoxia, crosstalk between AMPK and HIF has often been suggested. An interrelated regulation of both mediators could help to orchestrate their concerted adaptation reaction. It has been reported that AMPK regulates transport mechanisms on the protein level, which are also targets for HIF1 on the transcriptional level, e.g., Na^+^/K^+^-ATPase, facilitated glucose transporters (GLUT) or Na^+^-coupled glucose transporter (SGLT1) and thus protects the cells from hypoxic damage [38,42,44,99].

As already mentioned above (Section 5.5), the HIF-P4Hs were suggested as a common regulation mechanism coupling both pathways under hypoxia. However, definitive proof for an activation of AMPK via HIF-P4Hs remains elusive. Nevertheless, many studies identified the formation of ROS under hypoxia as the starting point for an inhibition of the HIF-P4Hs and a subsequent activation of HIF [71,100,101,102], while AMPK could be activated via subsequent increase in [Ca^2+^_i_] and CaMKK2 (see Section 5.4.) [103]. Sallé-Lefort et al. [67] even showed an effect of CaMKK2 inhibition on HIF1α stabilization under hypoxia, which might imply a direct regulation of HIF1α via CaMKK2 or AMPK.

Besides, there are also other hints at a direct regulation of HIF1 by AMPK and vice versa. After inhibition or knockout of AMPK, the activation of HIF1 was blunted under hypoxia or even when the HIF-P4Hs were inhibited [103,104]. It has been reported before that phosphorylation of HIF1α was necessary for its transcriptional activity [105,106], and as a protein kinase, this would be the most obvious effect AMPK could have on HIF1α. This phosphorylation could either happen directly at HIF1α [100] or at upstream factors [104], regulating their inhibitory effects on HIF1α, e.g., by inhibiting hydroxylation of HIF [104].

In contrast, other studies report no crosstalk between AMPK and HIF, although their targets are redundant [5]. In either AMPK or HIF1α knockout cells, an independent activation of each was demonstrated, ruling out a direct activation of one by the other [107,108]. There are even studies indicating antagonistic regulation mechanisms, showing an upregulation of HIF1α when AMPK activity is reduced, at least in cancer cells [109,110,111]. This might be mediated by AMPK promoting HIF-P4H activity [112]. This fits well with the observation of AMPK ensuring high α-ketoglutarate levels, which is an important cofactor for the HIF-P4Hs [22].

The fact that this antagonistic regulation has mostly been observed in cancer cells so far draws attention to the roles AMPK and HIF1 are supposed to play in tumorigenesis. While AMPK is considered anti-carcinogenic, HIF1 is a pivotal factor in ensuring tumor survival and initiation of the Warburg effect [111]. This might be illustrated by these findings of an antagonistic regulation, especially in cancer cells, although the role of AMPK in tumorigenesis is not yet completely clear, and it might also enhance tumor survival similarly or complementary to HIF1 [5].

Beyond differences between cancer and normal cells, the effects of AMPK might generally be tissue-specific due to different metabolic profiles and needs. Majd et al. [113] reported varying time courses and intensities of AMPK activation under short-term ischemia in rat brain, liver, kidney and heart that coincide with the individual tissue sensitivities to ischemic insults. This tissue specificity might be mediated by the different combinations of AMPK subunit isoforms [8] whose specific roles remain elusive for the most part to date [114] and might mirror the metabolic specificities of different tissues. Additionally, interspecies differences have been observed [114] that will make elucidating the tissue-specific distribution and function of the different compositions of AMPK even more challenging.

Taken together, there are several pros, as well as cons, regarding a crosstalk between AMPK and HIF. Research on this topic should be intensified in order to get a better idea of the mechanisms connecting them.

Apart from classic hypoxic signaling, AMPK is linked to metabolic signaling on several levels. The AMPK/sirtuin (SIRT) 1/peroxisome proliferator-activated receptor-γ coactivator-1α (PGC-1α) axis is strongly involved in metabolic adaptation [115,116] and one of the better-known connections, although its role under hypoxia has not been clarified so far. The activation of PGC-1α, in turn, has been shown to downregulate nuclear factor κB (NFκB) [117,118], a transcription factor that is considered to link the immune response with the adaptation reaction to hypoxia [119,120]. Thus, the anti-inflammatory effect of AMPK might act as a feedback mechanism to avoid an over-activation of the immune system under cellular stress. In contrast, an activation of NFκB via TAK1, the putatively third upstream kinase of AMPK, has been described [121], also suggesting a positive relation of AMPK and NFκB activation or rather a context or tissue-dependent interplay of the various factors, fine-tuning the ideal reaction to different stressors.

## 7. Conclusions

AMPK is widely regarded to exert protective effects under hypoxia. A concerted action together with HIF would ensure cellular adaptation both in the short-term (by AMPK) and long-term (by HIF). Thus, AMPK could adapt cellular metabolism quickly after the onset of hypoxia and thus ensure cellular survival until the more sustainable adaptation mediated by HIF becomes effective. For instance, AMPK regulates transport mechanisms on the protein level that are transcriptional targets of HIF. An oxygen-dependent regulation could provide a timely activation of AMPK, so that it is activated even before cellular energy levels, and thus integrity, are affected.

This beneficial effect might be of clinical use in patients with increased risk for hypoxia-associated pathologies, like ischemic stroke or myocardial infarction, but also hypoxia-associated diseases like inflammatory bowel disease. With AMPK agonists, like the antidiabetic drug metformin, already being in clinical use their applications might easily be extended.

Altogether, there are manifold studies covering different aspects of AMPK regulation and its role and activation under hypoxia. However, the hypotheses and approaches to prove them are nearly as diverse as the number of studies. Thus, the current understanding of the regulation of AMPK activity under hypoxia is rather puzzling, and for a better understanding future studies should put special emphasis on characterizing the tissues and the tissue-specific AMPK isoforms under investigation, as well as excluding alternative pathways that might interact with the proposed mechanism.

## Abbreviations

AMPK	AMP-activated protein kinase
CaMKK	Ca^2+^/calmodulin-dependent protein kinase
DMOG	Dimethyloxalylglycine
FIH	Factor inhibiting HIF
HIF	Hypoxia-inducible factor
HIF-P4H	HIF-prolyl-4-hydroxylase
LKB	Liver kinase B
MDPI	Multidisciplinary Digital Publishing Institute
mTORC	Mammalian target of rapamycin complex
PGC-1α	Peroxisome proliferator-activated receptor-γ coactivator-1α
ROS	Reactive oxygen species
SIRT	Sirtuin
TAK	Transforming growth factor β-activated kinase
